# Influence of different treatment techniques on radiation dose to the LAD coronary artery

**DOI:** 10.1186/1748-717X-2-20

**Published:** 2007-06-05

**Authors:** Carsten Nieder, Sabine Schill, Peter Kneschaurek, Michael Molls

**Affiliations:** 1 Radiation Oncology Unit, Nordlandssykehuset HF, 8092 Bodø, Norway; 2 Department of Radiation Oncology, Klinikum rechts der Isar der Technischen Universität München, Ismaninger Str. 22, 81675 Munich, Germany

## Abstract

**Background:**

The purpose of this proof-of-principle study was to test the ability of an intensity-modulated radiotherapy (IMRT) technique to reduce the radiation dose to the heart plus the left ventricle and a coronary artery. Radiation-induced heart disease might be a serious complication in long-term cancer survivors.

**Methods:**

Planning CT scans from 6 female patients were available. They were part of a previous study of mediastinal IMRT for target volumes used in lymphoma treatment that included 8 patients and represent all cases where the left anterior descending coronary artery (LAD) could be contoured. We compared 6 MV AP/PA opposed fields to a 3D conformal 4-field technique and an optimised 7-field step-and-shoot IMRT technique and evaluated DVH's for several structures. The planning system was BrainSCAN 5.21 (BrainLAB, Heimstetten, Germany).

**Results:**

IMRT maintained target volume coverage but resulted in better dose reduction to the heart, left ventricle and LAD than the other techniques. Selective dose reduction could be accomplished, although not to the degree initially attempted. The median LAD dose was approximately 50% lower with IMRT. In 5 out of 6 patients, IMRT was the best technique with regard to heart sparing.

**Conclusion:**

IMRT techniques are able to reduce the radiation dose to the heart. In addition to dose reduction to whole heart, individualised dose distributions can be created, which spare, e.g., one ventricle plus one of the coronary arteries. Certain patients with well-defined vessel pathology might profit from an approach of general heart sparing with further selective dose reduction, accounting for the individual aspects of pre-existing damage.

## Background

Intensity-modulated radiation therapy (IMRT) can be used to reduce the dose to critical organs such as the heart in mediastinal radiotherapy [1-6]. This might impact on long-term side effects especially in highly curable diseases, e.g., in patients with Hodgkin's and non-Hodgkin's lymphoma [7-10]. The current study in female patients with target volumes typical for lymphoma treatment examines the ability of a previously developed IMRT technique [5] to spare not only the heart as a complete organ and its chambers, for example the left ventricle, but also another well-defined region within the heart, such as, a coronary artery. As recently suggested, arteries appear to be particularly vulnerable to the effects of ionising radiation [11]. Radiation of the endothelium might cause early functional alterations such as pro-inflammatory responses and other changes, which are slowly progressive and might interact fatally with atherosclerotic lesions. An IMRT plan optimisation that takes the localisation of a critical vessel into account in individuals with known, localised coronary artery stenosis might allow for selective dose reduction. The present study evaluates the feasibility of an already heart-sparing IMRT technique to create such an individualised dose distribution.

## Materials and methods

We used the original data set of 8 female patients that formed the basis for development of our heart-sparing IMRT technique to identify the most suitable coronary artery for this study, i.e. the artery that could be reliably contoured in as many patients as possible. Females were chosen because of the challenge to obtain low breast doses in addition to heart sparing. Our attempt to reliably identify one of the coronary arteries was successful in 6 of the 8 patients. The left anterior descending artery (LAD) could be contoured with the help of a radiologist and was therefore further explored for the purpose of this study. These 6 patients were older than the others and some of them had slight vessel calcifications, which facilitated delination.

**Figure 1:**
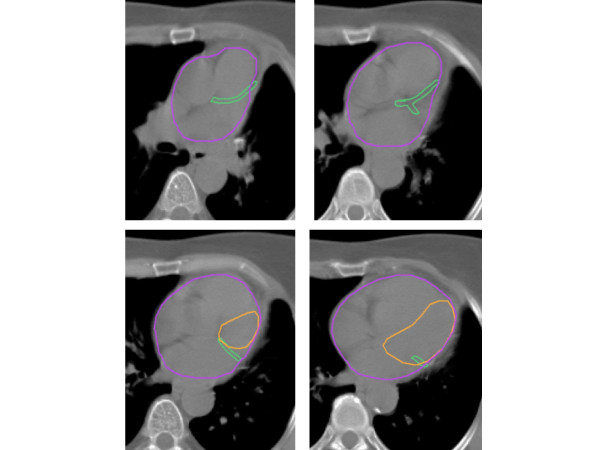
Treatment planning computed tomography scan with contoured left anterior descending coronary artery (and part of the left circumflex artery) in green color, left ventricle in orange and heart in purple.

**Figure 2:**
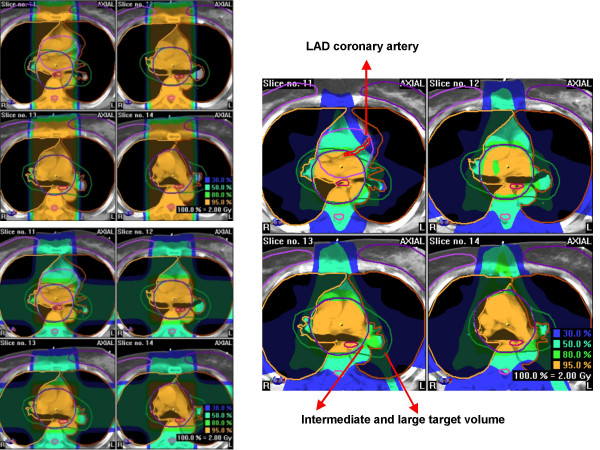
Treatment planning computed tomography scan with contoured organs at risk (incl. left anterior descending coronary artery in red color, on the small images in green color), clinical target volume (both intermediate and large scenario in the same patient) and isodose distributions for the intermediate scenario with ap-pa (upper left), 4-field (lower left), and 7-field IMRT technique (right) in the same patient.

As already described [5], the planning computed tomography (CT) scans were performed in standard supine position during free breathing. The CT scanner was a Siemens Somatom Plus4. The scans were performed with 8 mm slice-thickness, scanned without gap. No contrast media were administered. Three different clinical target volume (CTV) scenarios were studied. The first one included the paraclavicular and upper mediastinal lymph nodes (median size 749 ccm, range 566–860 ccm). Expanded CTV's also including a. the lower mediastinum (median size 1008 ccm, range 774–1337 ccm) and b. the lower mediastinum and both hilar regions (median size 1142 ccm, range 936–1664 ccm) were examined too. Figures 1 and 2 provide examples of target volumes and organs at risk. We contoured left and right lung, esophagus, spinal cord, breasts, heart, left ventricle and LAD.

As in the original 8 patients, the coplanar single-isocenter 7-field step-and-shoot IMRT technique developed by our group (gantry angles of 0, 51, 102, 153, 204, 255, and 306°) was compared with AP/PA opposed fields and a coplanar single-isocenter 4-field technique (beam angles 0, 180, 90, and 270°). A Siemens Mevatron KD-2 linear accelerator with a 58 leaf multi-leaf collimator and leaf width of 1 cm (6 MV photons) was used. The 7 IMRT fields consisted of 13–18 sub-segments each (median 15). The gantry angles remained unchanged for all 6 cases, i.e. no individual optimization was attempted. A dose of 2 Gy per fraction was chosen for a total dose of 30 Gy, reflecting current concepts in many types of lymphoma. These doses were prescribed to the isocenter. The PTV was to be surrounded by the 95% isodose line. In IMRT, 100% of the PTV was to receive 95% of the prescribed dose. The constraints for organs at risk in IMRT were chosen as follows: absolute maximum dose to the left ventricle 50% of the prescription dose (i.e. 1 Gy), dose to 25% of the volume 25% (0.5 Gy), dose to 50% of the volume 25% (0.5 Gy), dose to 75% of the volume 20% (0.4 Gy). The heart should receive an absolute maximum dose of 75% and not more than 20% to 70% of the volume, 40% to 40% of the volume, and 60% to 20% of the volume. The planning system used for all techniques and scenarios was BrainSCAN 5.21 (BrainLAB, Heimstetten, Germany), which uses a pencil beam algorithm and heterogeneity corrections. BrainSCAN offers the option of adjusting the priority of each organ at risk relative to the others by specifying organ at risk guardian values. We assigned equally high priority to the heart, left ventricle and LAD (guardian 100%), because patients with coronary artery disease will often have myocardial damage in addition. The calculation grid size was 4 mm.

We used the Kruskal-Wallis-test for global statistic evaluation of differences in PTV and organ at risk DVH's, followed by post hoc analysis with the Mann-Whitney test (all performed with the SPSS software). A p-value < 0.05 was considered statistically significant.

## Results

The two patients that could not be included in the present LAD study were relatively young and had a. the smallest PTV and heart from the original group of 8 patients and b. intermediate values for these two parameters, respectively. No other special anatomic features distinguished the non-eligible two cases from the eligible 6 cases.

### Dose to the heart and left ventricle

The results of DVH analysis remained essentially unchanged in the study group of 6 patients compared to the original group. The small target volume excluded most of the heart. Therefore, no difference between the 3 techniques could be observed [5]. For both other target volumes, the maximum doses were comparable. Nevertheless, IMRT resulted in better dose reduction to the heart and left ventricle than both other techniques. Better heart sparing was achieved when looking at the median dose, the volume receiving 30 Gy, i.e. 100% of the prescribed dose, and all dose levels down to the 15% isodose. The heart volume receiving 10% or less of the prescribed dose was similar for all techniques. Compared to IMRT with dose constraints only to heart and left ventricle, addition of LAD sparing had no consistent impact on heart DVH's, while high-dose areas in the left ventricle tended to be slightly reduced in 5 out of 6 patients. As discussed in the next paragraph, the main problem of adding the LAD as organ at risk was to maintain target volume coverage.

### Dose to the LAD coronary artery

**Figure 3:**
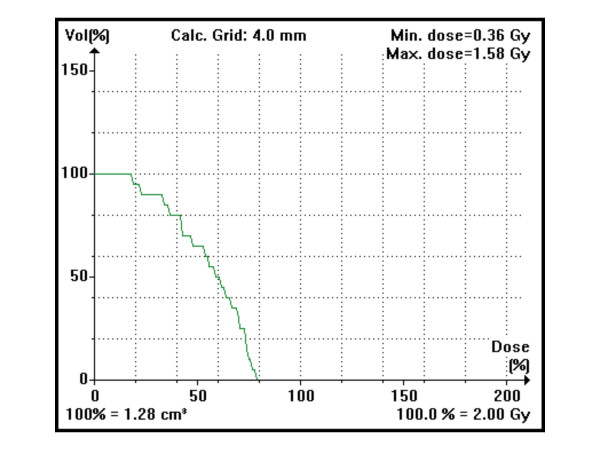
Dose-volume histogram for the left anterior descending coronary artery with the 7-field IMRT technique in the intermediate target volume scenario.

The median contoured volume was 1.94 ccm (range 1.28–2.86). As shown in Table 1, the AP/PA and the 4-field technique gave very similar results for most parameters, with some advantages for 4 fields in the intermediate and large target volume scenarios. Even with IMRT, high-dose areas could not be avoided completely, because the distance between PTV and LAD was too small (Figure 2). However, IMRT resulted in the lowest median LAD dose in all 3 scenarios and in the smallest volume of LAD receiving 100% of the prescribed dose. The median LAD dose was reduced by at least 44% with even larger reductions in the volume of LAD receiving 100% of the prescribed dose. The advantage of IMRT disappeared in most cases below or around the 25% isodose level. We stepwise tested several strong LAD dose constraints, still aiming at the desired PTV coverage. However, unacceptable PTV underdosage required the assignment of looser constraints. The strongest ones that were acceptable and finally used were a maximum dose of 60%, 20% dose to 75% of the LAD volume, 40% dose to 50% of the volume, and 50% dose to 20% of the volume. In all patients, even these relatively generous constraints were not exactly met. The typical failure consisted of higher maximum doses than 60%. Table 2 summarizes the results and optimal technique for each given patient and Figure 3 displays a typical dose-volume histogram.

**Table 1:** Median doses to the left anterior descending coronary artery (LAD) in [Gy] for 3 differently sized target volumes

	Maximum dose (range)*	Median dose (range)	Median volume receiving 100%	Median volume receiving 25%*
Small, AP-PA	28.5 Gy (27.3–29.4)	23.4 Gy (18.3–27.0)	0% (0-0)	69% (65–80)
Small, 4-field	29.7 Gy (28.5–30.0)	21.3 Gy (13.2–26.1)	0% (0-0)	72% (56–80)
Small, IMRT	28.5 Gy (21.3–29.7)	11.1 Gy (8.7–14.1)	0% (0-0)	70% (53–75)
Intermediate, AP-PA	30.6 Gy (30.0–31.2)	30.0 Gy (26.4–30.0)	50% (1–82)	98% (74–100)
Intermediate, 4-field	30.6 Gy (30.0–30.9)	29.0 Gy (19.2–30.3)	23% (0–93)	100% (89–100)
Intermediate, IMRT	26.9 Gy (23.7–30.0)	15.9 Gy (10.8–29.4)	0.25% (0–26)	92% (78–100)
Large, AP-PA	31.5 Gy (29.7–31.8)	30.5 Gy (30.2–30.6)	90% (60–98)	100% (100-100)
Large, 4-field	31.2 Gy (30.6–31.8)	28.5 Gy (21.9–29.4)	23% (18–25)	100% (100-100)
Large, IMRT	29.1 Gy (27.3–31.2)	15.9 Gy (15.0–21.9)	3% (0–9)	88% (82–95)

* Statistical testing was not performed for these parameters.

p < 0.01 for comparison of the median LAD dose with ap-pa vs. IMRT and 4-field vs. IMRT.

p < 0.01 for comparison of the median volume receiving 100% with ap-pa vs. both 4-field and IMRT (for both intermediate and large PTV). The differences between 4-field and IMRT are also statistically significant.

Small volume: upper mediastinal plus paraclavicular nodal areas (566–860 ccm), intermediate volume: lower mediastinal nodes in addition (774–1337 ccm), large volume: hilar nodes in addition (936–1664 ccm)

**Table 2:** Results for each of the 6 patients

Patient Nr.	Magnitude of IMRT advantage in LAD sparing for both intermediate and large target volume scenarios	Would a decision for IMRT have been the preferred option also with regard to heart and left ventricle sparing?
1	IMRT outperformed the other techniques to just below the 25% isodose	Yes, IMRT was optimal
2	IMRT outperformed the other techniques to just below the 25% isodose	Yes, IMRT was optimal
3	IMRT outperformed the other techniques to just below the 25% isodose	Yes, IMRT was optimal
4	IMRT outperformed the other techniques down to the 25% isodose	Yes, IMRT was optimal
5	IMRT outperformed the other techniques down to the 50% isodose	IMRT and 4-field were very similar regarding total heart, but IMRT was slightly better regarding median and mean left ventricle dose (maximum doses were similar, as were ≤25% isodose levels)
6	IMRT and 4-field very similar, both outperformed AP/PA down to the 50% isodose	No, 4-field was best (lowest median heart dose and volume receiving 2 Gy, no disadvantage regarding maximum dose and the various low- dose parameters)

LAD: left anterior descending coronary artery

### Dose to the other organs at risk and PTV

Compared to the results in the original group of 8 patients, the dose distributions in the other contoured organs at risk remained essentially unchanged. The same holds true for the finding of similar PTV coverage with all three techniques. Interestingly, the PTV was more sensitive than the organs at risk to introduction of strong LAD dose constraints.

## Discussion

The present extension of our systematic IMRT treatment planning study was performed in a very challenging patient population, i.e. females with different sizes of paraclavicular and mediastinal target volumes and presumed cardiac disease, necessitating selective sparing of vulnerable structures within the heart. The IMRT technique was previously developed and optimized with regard to beam angles and dose constraints by our group [5]. We found during this process, that 7 equally-spaced beams resulted in satisfactory PTV coverage, dose homogeneity, and adherence to the normal tissue constraints. Other groups have also shown that 7-field IMRT can be a useful technique in this region of the body, e.g. in esophageal cancer [1,2].

The aim of general heart sparing was best achieved with IMRT. Yet, high doses to some parts of the heart will still occur if the distance between the heart and PTV is marginal or even absent. It is certainly important whether such high-dose areas are located in regions with better or compromised perfusion. Therefore we decided to perform this proof-of-principle-study aiming at selective protection of a well-defined small substructure. With the available CT equipment, the LAD was the most suitable structure, which could be contoured in 6 patients. It should be noted that cardiac CT imaging protocols that use, e.g., a smaller slice thickness, would enable us to depict longer segments of the coronary tree and smaller branches [12]. In addition, the vessel contours could be delinated more sharply and a higher contrast-to-noise ratio could be obtained. Therefore, our LAD contours might underestimate the true extent of the vessel. Irrespective of scanner parameters, the small branches will eventually disappear within the myocardium of the left ventricle, which is feeded by these branches and which was also considered as organ at risk. In our study, IMRT resulted in the lowest median LAD dose in all 3 scenarios and in the smallest volume of LAD receiving 100% of the prescribed dose and eventually provided the most suitable plan in 5 out of 6 patients. In IMRT with dose constraints to the heart and left ventricle only, maximum doses of 113% in the LAD occurred [5]. In the present study, the maximum was reduced to 106%. But more importantly, the LAD volume receiving doses ≥100% was smaller and a pronounced sparing from intermediate doses could be obtained.

To our knowledge, no firm human data allow us to answer the question of which doses are most damaging to the coronary arteries, i.e. the "a lot to a little or a little to a lot" question. Intuitively, and supported by the data discussed by Schultz-Hector and Trott [11], one would like to reduce the whole area under the DVH as much as possible, but in addition obtain pronounced reductions in the high-dose regions, because it can not be assumed that a coronary artery reacts like a parallel organ. Whether the significant improvements in dose distribution by IMRT translate into clinical benefits, requires prospective confirmation. In addition, the individual magnitude of benefit from selective dose-reduction might depend on the extent of pre-existing damage. Optimal planning of such individualised dose distributions beyond a proof-of-principle study will require more information than that provided by standard-CT. Angiography, cardio-CT and/or magnetic resonance imaging will likely have to add data on individual patient anatomy and pre-existing damage. Another important question is how reliably such individual dose distributions can be transferred into daily routine, where breathing, swallowing, cardiac motion and set-up errors need to be taken into account. Therefore, assessment of the influence of motion artefacts and the need for definition of a safety margin around the LAD is necessary. It might be possible to use virtual volumes to protect small structures at risk, as described by Girinsky et al. [4] who found this strategy superior to dose constraints assigned to individual organs. However, a detailed discussion of gating, high-precision and 4-D radiotherapy methodology [13,14], is beyond the scope of this article.

As described earlier, there is a price to pay for optimized heart sparing with IMRT compared to AP/PA: both a higher mean lung dose (of lesser concern with a total dose of only 30 Gy) and higher exposure of breast parenchyma to low radiation doses [5]. However, both the mean lung dose, V20 and V30 is not higher with IMRT than with 3-D 4 fields, which is important for patients with non-lymphoma mediastinal malignancy, where AP/PA techniques clearly are inappropiate because the total doses required are much higher than 30 Gy. The IMRT disadvantages described in our patients were also found in the plan comparisons by Girinsky et al. [4]. It appears therefore necessary to select very carefully the patients where heart sparing is of utmost importance and to weigh the benefits against the disadvantages. Further refinement is hoped to result from continued optimization of target volume concepts, e.g., based on positron emission tomography and early response evaluation during chemotherapy [15,16], as toxicity risks will decrease with further reduction of the target volumes [17]. Proton treatment of thoracic target volumes appears to reduce the dose to normal tissues significantly, compared with photon therapy, either 3D-conformal or IMRT [18]. However, no planning studies of selective dose reduction to certain substructures of the heart have yet been performed and the issue of precise delivery of such plans to patients is not less complicated in proton radiotherapy.

## Conclusion

The 7-field IMRT technique provided better heart sparing than traditional approaches in the majority of patients. In addition to dose reduction to the whole organ, individualised dose distributions can be created, which spare, e.g., one ventricle plus one of the coronary arteries. Certain patients with well-defined vessel pathology might profit from an approach of general heart sparing with further selective dose reduction, accounting for the individual aspects of pre-existing damage.

## Competing interests

The author(s) declare that they have no competing interests.

## Authors' contributions

CN and MM participated in the conception and design of the study and the target volume definition. SS and PK created the treatment plans and performed data acquisition. CN and SS performed data analysis and interpretation and drafted the manuscript. All authors read and approved the final manuscript.
